# A Novel Photosynthesis of Carboxymethyl Starch-Stabilized Silver Nanoparticles

**DOI:** 10.1155/2014/514563

**Published:** 2014-01-29

**Authors:** M. A. El-Sheikh

**Affiliations:** National Research Centre, Textile Research Division, El-Behouth Street, Dokki, P.O. Box 12311, Giza, Egypt

## Abstract

The water soluble photoinitiator (PI) 4-(trimethyl ammonium methyl) benzophenone chloride is used for the first time in the synthesis of silver nanoparticles (AgNPs). A new green synthesis method involves using PI/UV system, carboxymethyl starch (CMS), silver nitrate, and water. A mechanism of the reduction of silver ions to AgNPs by PI/UV system as well as by the newly born aldehydic groups was proposed. The synthesis process was assessed by UV-vis spectra and TEM of AgNPs colloidal solution. The highest absorbance was obtained using CMS, PI and AgNO_3_ concentrations of 10 g/L, 1 g/L, and 1 g/L, respectively; 40°C; 60 min; pH 7; and a material : liquor ratio 1 : 20. AgNPs so-obtained were stable in aqueous solution over a period of three weeks at room temperature (~25°C) and have round shape morphology. The sizes of synthesized AgNPs were in the range of 1–21 nm and the highest counts % of these particles were for particles of 6–10 and 1–3 nm, respectively.

## 1. Introduction

Silver nanoparticles have unique optical, electrical, and biological properties that have attracted significant attention due to their potential use in many applications, such as catalysis, biosensing, drug delivery, and medical textiles [1–5].

Techniques used in the formation of nanosized metal particles are Top-down and Bottom-up techniques. Top-down is mechanically based on diminishing processes that start from bulk metals. Bottom-up technique predominantly employs the chemical or electrochemical reduction of metal salts in solution. Thermolytic, photolytic, radiolytic, laser irradiation, microwave heating, or sonochemical procedures, as well as the decomposition of appropriate metastable precursor molecules, complete the wide spectrum of synthetic pathways to metal nanoparticles. The formation of nanoparticles occurs when individual, neutral atoms are subjected to collide with other atoms, resulting in a nucleation to smaller or larger particles [6].

Sodium borohydride, hydroxylamine hydrochloride, dimethylamine borane, sodium citrate, hydrazine monohydrate, sodium formate, trimethylamine borane, sodium trimethoxyborohydride, and formaldehyde have all become routine reducing agents for the generation of metal NPs. Among the various reducing agents, BH^4−^ plays the dominant role. These chemicals are highly reactive and cause potential environmental and biological risks [7, 8]. Utilization of nontoxic chemicals, environmentally gentle solvents and renewable materials is one of the key issues that worth important consideration in a green synthesis policy.

Direct photoreduction and photosensitization are powerful approaches for the in situ synthesis in polymer matrixes [9–16]. The heart of the photochemical approach is the generation of M^0^ (metal nanoparticles) in such conditions that their precipitation is thwarted. Metal nanoparticles can be formed through direct photoreduction of a silver source, silver salt, or complex, or reduction of silver ions using photochemically generated intermediates, such as radicals. The photoreduction is often promoted by dyes dispersed or dissolved in the polymer or present in the chemical structure of the matrix. The visible light photoinitiator “camphorquinone” was used to generate electron-donating radicals upon photolysis. Subsequent oxidation of these radicals to the corresponding cations in the presence of silver hexafluoroantimonate (AgSbF_6_) leads to the simultaneous formation of silver nanoparticles. The key step of the process is the reaction of silver cations with photogenerated transient species that are able to reduce them to silver metal atoms. Two classes of photoinduced reactions were used to produce these primary radicals. The first one is based on the reaction of an electron rich molecule (e.g., amine, thiol, and ether) with the highly oxidant triplet state of a sensitizer excited upon absorption of the actinic photons. The second one involves the direct hemolytic photocleavage of a sigma bond (mainly C–C bonds adjacent to a carbonyl) [17].

Since the polymers prevent agglomeration and precipitation of the particles, they have been frequently employed as capping or stabilizing agents in the chemical synthesis of metal nanoparticles. Among methods of green synthesis of silver nanoparticles, water is used as an environmentally benign solvent and polysaccharides as a capping agent, or in some cases polysaccharides serve as both a reducing and a capping agent [4, 8, 18–27]. In a case of dual polysaccharide function, silver nanoparticles were synthesized by the reduction of Ag^+^ inside of nanoscopic starch templates. The extensive network of hydrogen bonds in the templates provides surface passivation or protection against nanoparticle aggregation [26]. Biosynthesis is also an alternative green technique for synthesis of silver nanoparticles [2, 28–31].

The so-called “alcohol reduction process” is a very general process for the production of metal nanoparticles, often stabilized by organic polymers. In general, the alcohols which were useful reducing agents contained *α*-hydrogen and were oxidized to the corresponding carbonyl compounds. The oxidation of primary alcohols (R–CH_2_OH) by Ag^+^ is also well established; the reaction is slow and requires heating to be accelerated as follows:

(1) $$\begin{array}{c} 2\text{A}{\text{g}}^{+}+\text{R–C}{\text{H}}_{2}\text{OH}⟶2\text{A}{\text{g}}^{0}+2{\text{H}}^{+}+\text{R–CHO} \end{array}$$

However, such a reaction is a very slow process and constitutes a minor route for Ag^+^ reduction in comparison with the photoinduced generation of AgNPs described hereafter [11, 32].

Bottom et al. [33, 34] developed and assessed the efficiency of a novel, water-soluble photosensitizer, 4-(trimethyl ammonium methyl) benzophenone chloride, and used it for the photoinitiated graft copolymerization of 2-hydroxyethyl acrylate onto cellulose. El-Sheikh and coworkers [35–38] further utilized this efficient photoinitiator in the graft copolymerization of acrylic acid and acrylamide onto carboxymethyl starch and in the photooxidation of starch. The above cited references are the only ones found in the literature about the using of 4-(trimethyla mmonium methyl) benzophenone chloride. To the best of the author's knowledge and according to literature survey, the later has never been used in the photosynthesis of AgNPs.

Benzophenone compounds are powders. They can absorb and dissipate UV radiation. Benzophenone compounds are used in bath products, makeup products, hair products, sunscreens, and skin care products. They protect the skin from the harmful effects of the sun.

According to the literature survey, one can describe the process as a “novel” process. According to the use of the benzophenone compounds in cosmetics, one can describe the photoinitiator used in this research work as a safe, ecofriendly, and green compound.

In this work, a novel green method was adopted to synthesize AgNPs using the water soluble 4-(trimethyl ammonium methyl) benzophenone chloride/UV system and silver nitrate as a precursor in the presence of a water soluble polymer (CMS). While PI photogenerate AgNPs, CMS act as both a reducing and stabilizing agent. All components are water soluble, the process is “simple and easy,” and the synthesis conditions used are mild. Parameters affecting the synthesis of AgNPs such as PI, silver nitrate, and CMS concentrations as well as reaction time and temperature are studied. The synthesized AgNPs were assessed by measuring the absorbance of the colloidal solution, the size, the shape, and the stability of the synthesized AgNPs.

## 2. Experimental

### 2.1. Materials

Native maize starch (St.) was kindly supplied by the Egyptian Company for Starch and Glucose Manufacture, Cairo, Egypt.

4-(Trimethyl ammonium methyl) benzophenone chloride, a water soluble photoinitiator that belongs to the benzophenone series (UV Absorbers), was supplied by “The associated Octel Ltd., Widnes, UK,” and used without further purification.

Monochloroacetic acid, sodium hydroxide, sodium carbonate, silver nitrate, hydrochloric acid, acetic acid, ethanol, and isopropanol were laboratory grade chemicals.

### 2.2. Equipment

The irradiation reaction vessel consists of a quick fit water-cooled 125 W medium-pressure Hg lamp assembly as a UV irradiation source immersed in a quick fit 150 mL cylindrical tube. The total dose of the UV irradiation was controlled by controlling the time of exposure, that is, the reaction time. The reaction temperature was controlled using a thermostatic water bath.

### 2.3. Method

#### 2.3.1. Carboxymethylation

Water soluble carboxymethyl starch with DS = 0.2 was prepared according to a reported method [39]. In this method, 100 g of maize starch was placed in a sealable bottle and mixed together with a known volume of isopropanol. An aqueous solution of sodium hydroxide (0.5 mole/mole St.) was added dropwise to the starch—isopropanol mixture under stirring until the whole amount of sodium hydroxide was added. The sodium salt solution of monochloroacetic acid (0.2 mole/mole St.), prepared by the reaction of monochloroacetic acid with the equivalent amount of sodium carbonate, was added dropwise to the starch—isopropanol—sodium hydroxide mixture under continuous stirring until the complete addition of the sodium monochloroacetate solution. Stirring was then stopped and the bottle was closed and kept at 30°C for 24 hours. After carboxymethylation, the CMS samples were washed with ethanol : water solution (80 : 20) while excess alkali was neutralized using acetic acid. After washing, the CMS samples were filtered and oven-dried at 70°C.

#### 2.3.2. Preparation of Silver Nanoparticles

In a beaker, a known weight of CMS was mixed with certain volume of distilled water using a mechanical stirrer. After complete dissolution of CMS, an aqueous solution of PI was added to the CMS solution followed by adding silver nitrate solution under continuous stirring until complete mixing of the whole contents. Doing so, the beaker contents were poured in the irradiation tube and transferred to a thermostatic water bath with a magnetic stirrer. The UV lamp is now immersed in the solution to just above the bottom of the tube to allow the magnet to move and to allow the whole solution to be exposed to the UV irradiation. The temperature was then allowed to rise gradually until the required temperature is reached. Finally, the UV lamp is switched on and the whole contents were kept at the synthesis temperature for a known period of time under continuous stirring. After synthesis, the yellowish brown colloidal solution is kept in a sealable bottle at room temperature (25°C).

### 2.4. Characterizations and Analyses

The DS of the carboxymethylated starch samples was determined via the determination of the carboxyl content according to a reported method [40].

The UV-vis spectra of silver nanoparticles and PI were recorded using UV-2401, UV-vis Spectrophotometer, Shimadzu, Japan, at wavelength range from 190 to 550 nm. *λ*max⁡⁡ at 256 nm is characteristic of PI, whereas *λ*max⁡⁡ at 390–420 is characteristic of silver nanoparticles. Synthesis of silver nanoparticles is expressed as the absorbance of the colloidal solution of the samples under test. The absorbance, the broadening, and the wavelength of the band measure the intensity of the colloidal solution, that is, the conversion of silver ions to AgNPs. Very concentrated AgNPs samples did not show one smooth band (either sharp or broad) but showed a number of crowded sharp bands. This behavior leads to false readings. So, concentrated samples which showed this behavior were diluted “*x*” times and the obtained absorbance value was then multiplied by “*x*” to obtain the actual absorbance value. The accuracy of this dilution technique was tested by comparing the absorbance readings of certain samples with moderate concentration before and after dilution. The readings were found approximately the same after multiplying by “*x*” times of dilutions.

Transmission Electron Microscope was used to characterize AgNPs. Thus, the shape and size of the synthesized silver nanoparticles were characterized by means of a JEOL-JEM-1200 Transmission Electron Microscope. The samples were prepared by placing a drop of the colloidal solution on a 400-mesh copper grid coated by an amorphous carbon film and evaporating the solvent in air at room temperature. The average diameter of the silver nanoparticles was determined from the diameter of nanoparticles found in several chosen areas in enlarged microphotographs. The particle size was measured from the TEM image using the software “Revolution v1.6.0b195,” a simple electron microscope tool for acquiring images, maps, and spectra using the Spectral Engine, 1999–2002 4pi analysis, Inc.

Stability of the silver nanoparticles was tested by storing samples in sealed glass bottles in a dark place at room temperature (~25°C) and then recording the spectra of the colloidal solutions after one week, two weeks, and three weeks of storing using UV-vis Spectrophotometer.

## 3. Results and Discussions

### 3.1. Mechanism of Photosynthesis of AgNPs

**Scheme 1:**
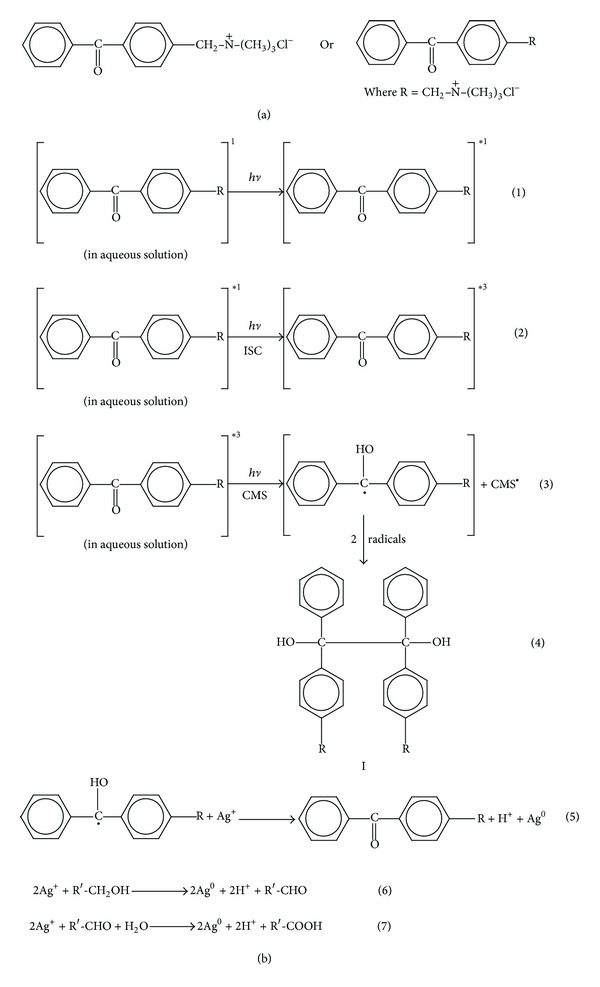
(a) Chemical structure of PI. (b) Mechanism of photosynthesis of AgNPs.

4-(Trimethyl ammonium methyl) benzophenone chloride structure is represented in Scheme 1(a). Under UV light excitation, 4-(trimethyl ammonium methyl) benzophenone chloride (in an aqueous medium) is first promoted to its excited singlet state (1); then, via fast intersystem crossing (ISC), it converts into triplet state (2). In the presence of CMS, which acts as a H-donor, the triplet transient state can undergo hydrogen abstraction with CMS to yield reactive radical species (3). Inactive species formation could take place by the combination of two radicals of PI (4) forming the pinnacol derivative (I) (Scheme 1(b)). The greater the extent of formation of compound I is, the more pronounced the inactivation of the photosynthesis process is [35–37]. The formation of CMS^•^ radical (3) initiates a photooxidation reaction which leads to the generation of new aldehydic end groups in the CMS chain. The hydroxyl and carboxyl groups originally present in the CMS molecules in addition to the newly generated aldehydic end groups, due to the photooxidation reaction, all can act as reducing groups of the Ag^+^ to Ag^0^ ((6) and (7)). In addition, the radical of the PI formed according to (3) can further reduce the silver ions to AgNPs as shown in (5). Accordingly, one can expect a very efficient reducing system that arose from (a) reducing groups of CMS, molecules, (b) radicals of CMS and (c) radicals of PI. At the same time while the reduction is keeping on and AgNPs grow gradually, CMS will further form a stable protection layer on the AgNPs surface.

Factors affecting the reduction and stability as well as the shape and size of the formed AgNPs along with reaction mechanism are given below.

### 3.2. Effect of Photoinitiator Concentration on the Formation of Silver Nanoparticles

**Figure 1:**
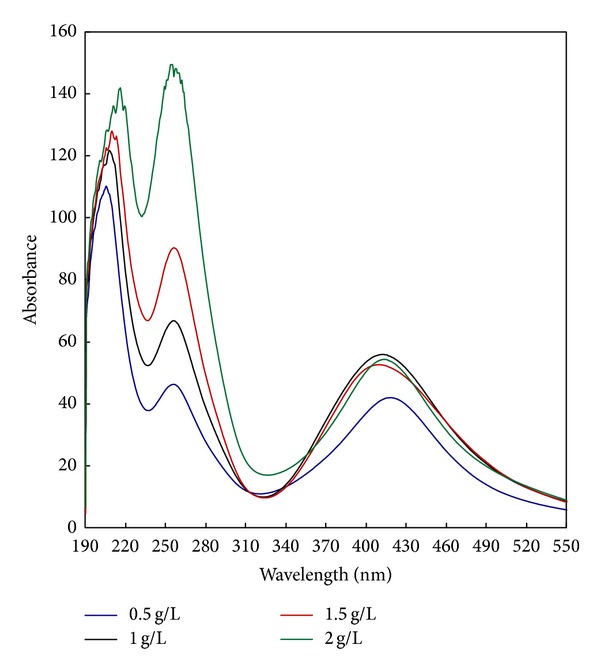
Effect of photoinitiator concentration on the synthesis of silver nanoparticles [CMS], 50 g/L; [PI], 0.5–2 g/L; [AgNO_3_], 1 g/L; temp., 30°C; time, 60 min.; M : L ratio, 1 : 20; pH, 7.

Different concentrations of PI (from 0.5 to 2 g/L) were used to study the effect of increasing the concentration of PI on the extent of photosynthesis of AgNPs. Thus, photosynthesis was carried out using PI/UV system for one hour of UV irradiation at 30°C using CMS and AgNO_3_ concentrations of 50 g/L and 1 g/L, respectively, and a material : liquor ratio (M : L ratio) 1 : 20 at pH 7. The dependence of the absorbance of the colloidal solution of the synthesized AgNPs on the concentration of PI is shown in Figure 1. Figure 1 shows that, regardless of the concentration of PI, four intensive bands with symmetrical bell shapes and high absorbance values at 390–420 nm are formed. This indicates the formation of AgNPs [26, 41] and reflects the efficiency of the current system in synthesizing AgNPs at the synthesis conditions. The results obtained from Figure 1 shows also that increasing the concentration of PI from 0.5 to 1 g/L is accompanied by a dramatic increase in the absorbance of the colloidal solution. Maximum absorbencies (at *λ* = 410 nm) of the colloidal solutions of AgNPs synthesized using 0.5 g/L and 1 g/L were 40.9 and 55.8, respectively. Incorporation of PI into the PI/UV system enhances the formation of CMS^•^ radical (3). Consequently, initiates a photooxidation reaction resulting in the generation of new aldehydic end groups in the CMS chain. In their turn, the newly born aldehydic groups reduce the Ag^+^ to Ag^0^. At the same time, the formation of PI^•^ radical (3) and the interaction of this radical with AgNO_3_ (5) can further reduce silver ions to AgNPs. Further increase in PI concentration from 1 to 2 g/L was accompanied by marginal decrease in the absorbance (nearly leveling off state is achieved). This could be due to the formation of the pinnacol derivative (I) (4) which, in its turn, leads to the inactivation of the photosynthesis of AgNPs.

The four sharp bands at 256 nm are representing the PI. As is clear (Figure 1), the intensity of the bands increases with increasing the concentration of PI from 0.5 to 2 g/L reflecting a steady state of concentration of PI. The consumption of the PI in formation of PI^•^ radical (3) or the pinnacol derivative (I) (4) is amended by the reformation of PI (5) according to its “life circle” shown in (1)–(5).

### 3.3. Effect of Silver Nitrate Concentration on the Synthesis of Silver Nanoparticles

**Figure 2:**
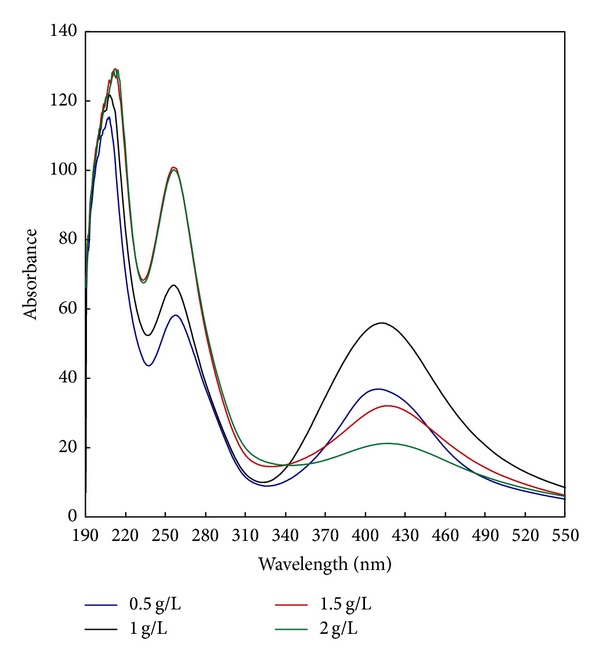
Effect of silver nitrate concentration on the synthesis of silver nanoparticles [CMS], 50 g/L; [PI], 1 g/L; [AgNO_3_], 0.5–2 g/L; temp., 30°C; time, 60 min.; M : L ratio, 1 : 20; pH, 7.

To study the effect of increasing the concentrations of AgNO_3_ on the photosynthesis of AgNPs, different concentrations of AgNO_3_ (from 0.5 to 2 g/L) were used. Thus, photosynthesis was carried out using PI/UV system for one hour of UV irradiation at 30°C using CMS and PI concentrations of 50 g/L and 1 g/L, respectively, and a M : L ratio 1 : 20 at pH 7. The dependence of the absorbance of the colloidal solution of the synthesized AgNPs on the concentration of AgNO_3_ is shown in Figure 2. It shows three intensive bell shaped bands at AgNO_3_ concentration of 0.5 g/L, 1 g/L, and 1.5 g/L. The fourth band representing AgNO_3_ concentration of 2 g/L band is broad without the bell shape characteristics. The figure also shows that the absorbance of the colloidal solution increases by increasing the concentration of AgNO_3_ from 0.5 to 1 g/L after which it decreases. It seems from the above findings that incorporation of PI into the PI/UV system in a concentration of 1 g/L enhances the formation of CMS^•^ radical and PI^•^ (3) in an amount just enough for the conversion of the Ag^+^ to Ag^0^ when the concentration of AgNO_3_ is in the range of 0.5–1 g/L. More reducing groups and more radicals are required to guarantee the conversion of all Ag^+^ to Ag^0^ when AgNO_3_ concentration is to be increased to more than 1 g/L. This could be achieved by increasing both PI and AgNO_3_ concentration together in a parallel way. It is also likely that higher concentration of AgNO_3_ acts in favor of agglomeration of the silver particles rather than in the formation of capped silver nanoparticles in a colloidal solution [5].

### 3.4. Effect of Carboxymethyl Starch Concentration on the Synthesis of Silver Nanoparticles

**Figure 3:**
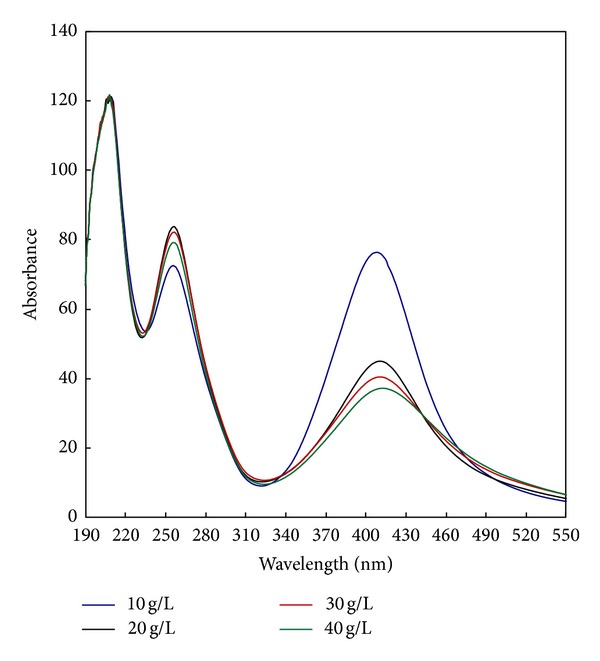
Effect of carboxymethyl starch concentration on the synthesis of silver nanoparticles [CMS], 10–40 g/L; [PI], 1 g/L; [AgNO_3_], 1 g/L; temp., 30°C; time, 60 min.; M : L ratio, 1 : 20; pH, 7.

Figure 3 shows the UV-vis absorption spectra of the colloidal solution of AgNPs prepared using different concentrations of CMS (10–40 g/L). The data reveal that, regardless of the CMS concentration used, four similar bands are formed at the four concentrations of CMS with a symmetrical bell shape which is characteristic of the formation of AgNPs. It is clear also that there is a gradual decrease in the absorption intensity by increasing the CMS concentration from 10 to 40 g/L. It should be mentioned that the least amount of CMS in the reaction medium (10 g/L) together with 1 g/L of PI is enough for full reduction of the Ag^+^ to Ag^0^ and efficient for stabilization of the formed AgNPs. The decrease in the absorbance as a result of increasing the concentration of CMS could be ascribed to the very concentrated mixture which decreases the mobility of PI and AgNO_3_ and hinders the conversion of Ag^+^ to Ag^0^.

### 3.5. Effect of Irradiation Time on the Synthesis of Silver Nanoparticles

**Figure 4:**
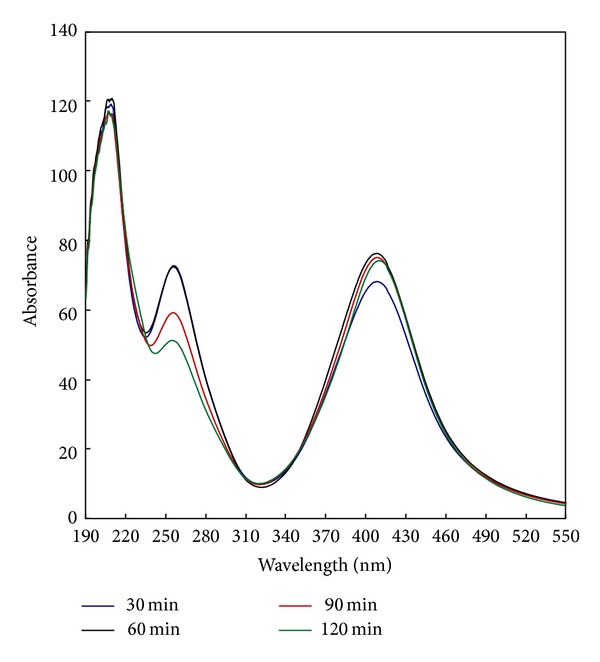
Effect of irradiation time on the synthesis of silver nanoparticles [CMS], 10 g/L; [PI], 1 g/L; [AgNO_3_], 1 g/L; temp., 30°C; time, 30–120 min.; M : L ratio, 1 : 20; pH, 7.

The irradiation dose was controlled by controlling the irradiation time; this is also the time of the photosynthesis reaction. A study of the irradiation time took place using irradiation duration from 30 to 120 min. The dependence of the absorbance of the colloidal solution of the synthesized AgNPs on the irradiation time is shown in Figure 4. Results in Figure 4 reveal that, regardless of irradiation time, four similar bands are formed at the four irradiation durations with a symmetrical bell shape which is characteristic of the formation of AgNPs. It can also be seen from the figure that increasing the irradiation time from 30 to 60 min is accompanied by an increase in the absorbance. It is also clear that absorbance values at 60–120 min are nearly the same showing a state of leveling off. The favorable effect of increasing the irradiation time on the synthesis of AgNPs is due to the formation of PI^•^ radicals which, in turn, produce the CMS^•^ radicals by H-abstraction from CMS. The role of these radicals in the conversion of Ag^+^ to Ag^0^ is clearly described in Section 3.2. Leveling off at 60–120 min means that no more conversion to silver nanoparticles will take place at the set of reaction conditions. It should be mentioned that 60 min of irradiation is enough for full reduction of the Ag^+^ to Ag^0^ nanoparticles.

### 3.6. Effect of Reaction Temperature on the Synthesis of Silver Nanoparticles

**Figure 5:**
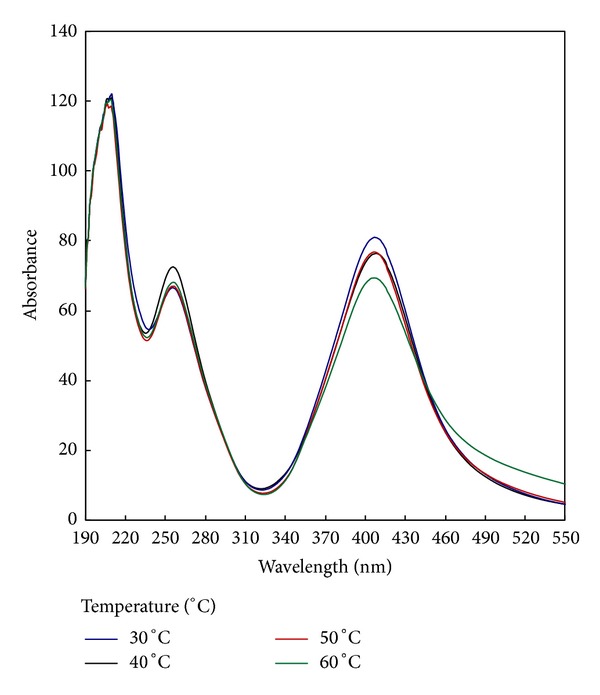
Effect of reaction temperature on the synthesis of silver nanoparticles [CMS], 10 g/L; [PI], 1 g/L; [AgNO_3_], 1 g/L; temp., 30–60°C; time, 60 min.; M : L ratio, 1 : 20; pH, 7.

To study the effect of increasing the synthesis temperature on the photosynthesis of AgNPs, different temperatures (from 30 to 60°C) were applied. The dependence of the absorbance of the colloidal solution of the synthesized AgNPs on the synthesis temperature is shown in Figure 5. Results in Figure 5 show that, regardless of photosynthesis temperature, similar bands are formed at all the photosynthesis temperatures under study with a symmetrical bell shape which is characteristic of the formation of AgNPs. The results obtained from Figure 5 also show that increasing the photosynthesis temperature from 30°C to 40°C is accompanied by an increase in the absorbance followed by a decrease in the absorbance by raising the temperature to 50–60°C. Increasing the temperature favors the formation of PI^•^ radicals which, in turn, produce the CMS^•^ radicals by H-abstraction from CMS. The role of these radicals in the conversion of Ag^+^ to Ag^0^ is clearly described in Section 3.2. Further increase in the reaction temperature (50–60°C) favors the formation of the pinnacol derivative (I) according to (4). Consequently, the inactivation of the photosynthesis process is more pronounced.

### 3.7. Stability of Silver Nanoparticles Synthesized at Different Conditions after One Week, Two Weeks, and Three Weeks of Storing

**Figure 6:**
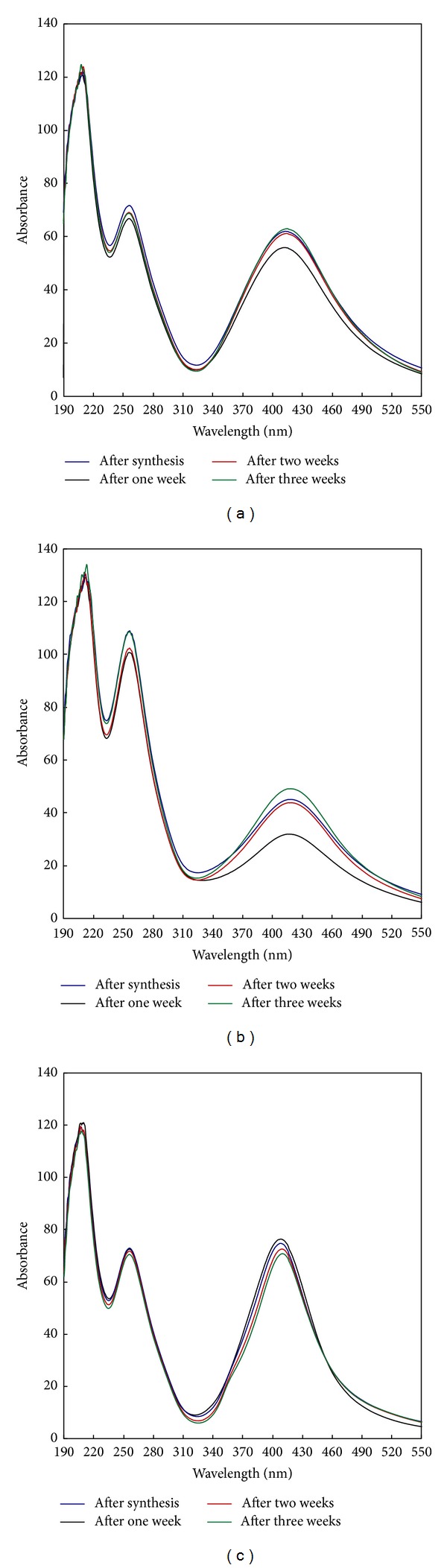
Stability of silver nanoparticles synthesized at different conditions after one week, two weeks, and three weeks of storing. (a) [CMS], 50 g/L; [PI], 1 g/L; [AgNO_3_], 1 g/L; temp., 30°C; time, 60 min.; M : L ratio, 1 : 20; pH, 7. (b) [CMS], 50 g/L; [PI], 1 g/L; [AgNO_3_], 1.5 g/L; temp., 30°C; time, 60 min.; M : L ratio, 1 : 20; pH, 7. (c) [CMS], 10 g/L; [PI], 1 g/L; [AgNO_3_], 1 g/L; temp., 30°C; time, 60 min.; M : L ratio, 1 : 20; pH, 7.

Stability of photosynthesized AgNPs was tested by measuring the absorbance of the colloidal solution after synthesis, after one week, after two weeks, and after three weeks of storing.  Figure 6 shows absorbance of the colloidal solution of samples synthesized at different conditions.

Figure 6 reveals that, regardless of storing duration and the set of the synthesis conditions, similar bands are formed for all samples with a symmetrical bell shape which is characteristic of the AgNPs.  Figure 6(a) shows that the absorbance curves and the absorbance maxima after one, two, and three weeks of storing are identical with an even higher absorbance value than that after synthesis. These data reveal that up to one week of storing, the reaction was continued at room temperature until all silver ions are converted to AgNPs followed by stability of the colloidal solution after such a long period of storing.  Figure 6(b) confirms the findings of Figure 6(a) that the lowest absorbance is related to the AgNPs of the freshly prepared sample and that storing even enhances the complete conversion of silver ions to AgNPs. In Figure 6(c), the case is different and the highest absorbance was related to the freshly prepared sample. Marginal decrease was noticed in the absorbance of samples stored for one, two, and three weeks, respectively. The decrease in the absorbance is very little and does not seem to affect the stability of the synthesized AgNPs. The only affecting parameter among samples a, b, and c is the concentration of CMS. CMS concentration in samples a and b is 50 g/L while it is 10 g/L for sample c. This may explain the continuity of the synthesis reaction for samples “a” and “b” but not for sample “c” where the initiation step successfully generated the CMS^•^ radical which, in its turn, initiates an oxidation reaction resulting in the generation of new aldehydic end groups capable of reducing Ag^+^ to Ag^0^. Although such a reaction is very slow and constitutes a minor route for Ag^+^ reduction at such a set of reaction conditions (room temperature and pH 7), the long storing time can finally lead to the formation of AgNPs.

### 3.8. TEM Micrograph and Particle Size Distribution Histogram of Silver Nanoparticles Synthesized at Different Synthesis Conditions

**Figure 7:**
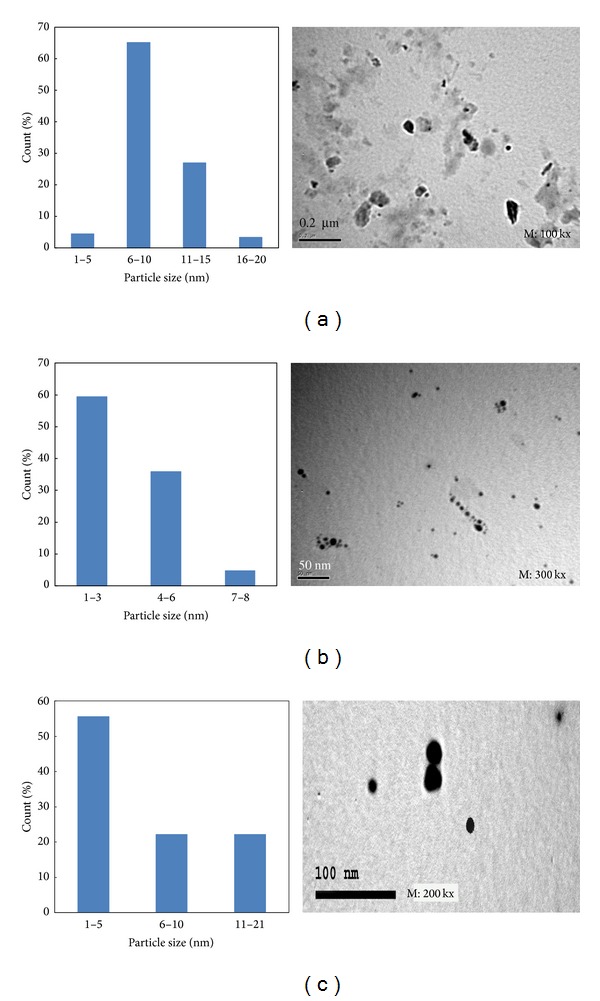
TEM micrograph and particle size distribution histogram of silver nanoparticles synthesized at different synthesis conditions. (a) [CMS], 50 g/L; [PI], 1 g/L; [AgNO_3_], 1 g/L; temp., 30°C; time, 60 min.; M : L ratio, 1 : 20; pH, 7. (b) [CMS], 50 g/L; [PI], 1 g/L; [AgNO_3_], 1.5 g/L; temp., 30°C; time, 60 min.; M : L ratio, 1 : 20; pH, 7. (c) [CMS], 10 g/L; [PI], 1 g/L; [AgNO_3_], 1 g/L; temp., 30°C; time, 60 min.; M : L ratio, 1 : 20; pH, 7.

The same AgNPs samples used for the stability test were again used to establish the TEM micrograph and particle size distribution histogram.  Figure 7 shows that, regardless of the reaction conditions, the three micrographs show that silver nanoparticles have round shape morphology and fine dispersion. Some aggregates were noticed in the micrograph of sample a. The mean particle sizes are between 1–20 nm, 1–8 nm, and 1–21 nm for samples a, b, and c, respectively. Some irregularly shaped particles were found in Figure 7(a) which could be due to aggregations of some nanoparticles. The highest count % was found for AgNPs between 6–10, 1–3, and 1–5 for samples a, b, and c, respectively.

## 4. Conclusions

4-(Trimethyl ammonium methyl) benzophenone chloride (a water soluble photoinitiator), PI/UV system, carboxymethyl starch (a water soluble ecofriendly polymer), and water (a solvent) were used to synthesis AgNPs using silver nitrate as a precursor. Thus, the chemicals and the process are green. The reduction of Ag^+^ to Ag^0^ was successfully achieved via the generation of active PI^•^ and CMS^•^ free radicals. Optimal reaction conditions that yield the highest absorbance values were CMS, PI, and AgNO_3_ concentrations of 10 g/L, 1 g/L, and 1 g/L, respectively, at 40°C for 60 min at pH 7 using a M : L ratio of 1 : 20. AgNPs so-obtained were stable in aqueous solution over a period of three weeks at room temperature (~25°C), and they have a round shape morphology. The sizes of synthesized AgNPs were found in the range of 1–21 nm and the highest counts % of these particles were for particles of 6–10 and 1–3 nm, respectively.
